# Metagenomic, (bio)chemical, and microscopic analyses reveal the potential for the cycling of sulfated EPS in Shark Bay pustular mats

**DOI:** 10.1038/s43705-022-00128-1

**Published:** 2022-05-19

**Authors:** Emilie J. Skoog, Kelsey R. Moore, Jian Gong, Davide Ciccarese, Lily Momper, Elise M. Cutts, Tanja Bosak

**Affiliations:** 1 Department of Earth, Atmospheric and Planetary Sciences, Massachusetts Institute of Technology, Cambridge, MA 02139 USA; 2 California Institute of Technology, Division of Geological and Planetary Sciences, Pasadena, CA 91125 USA; 3 Exponent, Inc., Pasadena, CA 91106 USA

**Keywords:** Microbial ecology, Biogeochemistry, Metagenomics, Biofilms, Next-generation sequencing

## Abstract

Cyanobacteria and extracellular polymeric substances (EPS) in peritidal pustular microbial mats have a two-billion-year-old fossil record. To understand the composition, production, degradation, and potential role of EPS in modern analogous communities, we sampled pustular mats from Shark Bay, Australia and analyzed their EPS matrix. Biochemical and microscopic analyses identified sulfated organic compounds as major components of mat EPS. Sulfur was more abundant in the unmineralized regions with cyanobacteria and less prevalent in areas that contained fewer cyanobacteria and more carbonate precipitates. Sequencing and assembly of the pustular mat sample resulted in 83 high-quality metagenome-assembled genomes (MAGs). Metagenomic analyses confirmed cyanobacteria as the primary sources of these sulfated polysaccharides. Genes encoding for sulfatases, glycosyl hydrolases, and other enzymes with predicted roles in the degradation of sulfated polysaccharides were detected in the MAGs of numerous clades including Bacteroidetes, Chloroflexi, Hydrogenedentes, Myxococcota, Verrucomicrobia, and Planctomycetes. Measurable sulfatase activity in pustular mats and fresh cyanobacterial EPS confirmed the role of sulfatases in the degradation of sulfated EPS. These findings suggest that the synthesis, modification, and degradation of sulfated polysaccharides influence microbial interactions, carbon cycling, and biomineralization processes within peritidal pustular microbial mats.

## Introduction

**Fig. 1:**
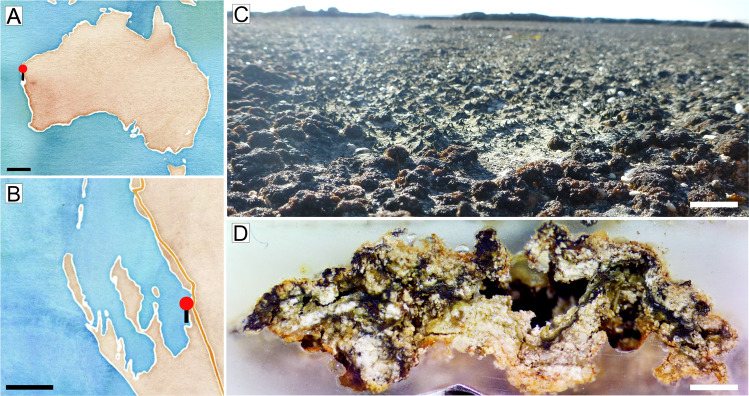
Peritidal pustular mats in Shark Bay, Western Australia. **A** Map showing the location of Shark Bay, Western Australia; **B** A closeup map of Shark Bay. Red pin shows Carbla Beach where pustular mats were collected. Maps were made using the folium package in Python; **C** Photograph of representative pustular mats (brown and red-brown structures). **D** Photograph of the cross-section of pustular mat from Shark Bay. The mat is not regularly laminated and has an uneven surface. Cyanobacteria are concentrated in the dark green-brown portions of the mat. The white regions contain sediment grains and precipitated minerals. Sediment grains are bound by EPS. Scale bars: 500 km (**A**), 50 km (**B**), 2.5 cm (**C**), 2 mm (**D**).

Silicified pustular marine microbial mats dating back to the Paleoproterozoic contain fossils of cyanobacteria embedded in EPS [1, 2]. Morphologically and ecologically analogous cyanobacteria can be found in modern marine pustular microbial mats in Abu Dhabi, Argentina, and other peritidal environments [3–5]. Perhaps the best studied contemporary analogs of these organisms reside in the hypersaline (~70 ppt) supratidal region of Hamelin Pool, Shark Bay, Australia [1, 6–8] (Fig. 1). There, the thick multilayered envelopes of EPS that coat cyanobacterial aggregates give shape to the distinctive pustules. EPS, which is thought to be central in the biogeochemical cycling within microbial mats [9–16], also enables the silicification of pustule-forming cyanobacteria [8, 17]. However, the connections between microbial community structure, functional potential, and activity within EPS and biogeochemical cycling of this organic matrix remain key unanswered questions in microbial mat ecology.

EPS can be a highly complex mixture of high-molecular-weight hydrated polysaccharides as well as proteins, extracellular DNA, and other noncarbohydrate constituents such as lactate, acetate, sulfate, phosphate, etc. bound to polymers [18–20]. Many marine algae and some benthic cyanobacteria from hypersaline environments are known to produce sulfated EPS [21–27]. A previous study reported sulfated polysaccharides produced by a sulfate reducing bacterium isolated from a microbial mat in The Bahamas and discussed the calcium-binding affinity of these compounds [28]. The degradation of these polymers requires various carbohydrate-active enzymes (CAZymes) and additional enzymes that remove various surface groups including sulfate [29]. Braissant et al. (2009) analyzed microbial mats from Eleuthera Island and demonstrated the activity of α-glucosidase, β-glucosidase, and β-galactosidase in the EPS, documented the abundance and variations in the acidity and charge of EPS extracted from different regions of these mats, and implicated the calcium binding capacity of this EPS in calcium carbonate precipitation [15]. A few more recent metagenomic and transcriptomic studies also reported carbohydrate metabolisms within microbial mats [11, 13, 14], but the links between microbial ecology and EPS production, modification, and degradation in these benthic systems remain to be established.

In this study, we address this by chemically mapping pustular mats from Shark Bay, Australia, showing that sulfur is particularly abundant in the regions of mat EPS that contain a strong chlorophyll autofluorescence signal, and confirming the presence of sulfated polysaccharides in the EPS matrix. Next, we assemble and analyze 83 microbial MAGs from pustular mats and identify microbes capable of producing and/or degrading sulfated and non-sulfated polysaccharides. Both metagenomic and biochemical analyses point to cyanobacteria as the main producers of sulfated polysaccharides. Metagenomic data also reveal the widespread potential of the microbial community in pustular mats to remove sulfate groups from sulfated EPS; sulfatase assays confirm this activity. Collectively, these analyses point to sulfated polysaccharides as a previously unrecognized component of pustular mats that likely influences physical properties, community structure, and biogeochemical cycling within these mats.

## Materials and methods

### Sampling

Pustular microbial mats were collected at Carbla Beach, Shark Bay, Western Australia (−26.141967, 114.236685) under Regulation 17 License #08-000373-1 in July 2017. Small samples of pustular mats, ~2.5 cm in diameter and ~5 mm thick, were excised by a sterile scalpel and stored in the dark on ice, in sterile 50 mL Falcon® centrifuge tubes filled with seawater. Samples were shipped on ice and transferred into sterile culture jars that contained hypersaline BG11 medium [30, 31] (10.17504/protocols.io.bkcmksu6). The present paper refers to these samples as the *original enrichment cultures* of pustular mats, in contrast to cyanobacterial enrichment cultures.

### Thin section microscopy

**Fig. 2:**
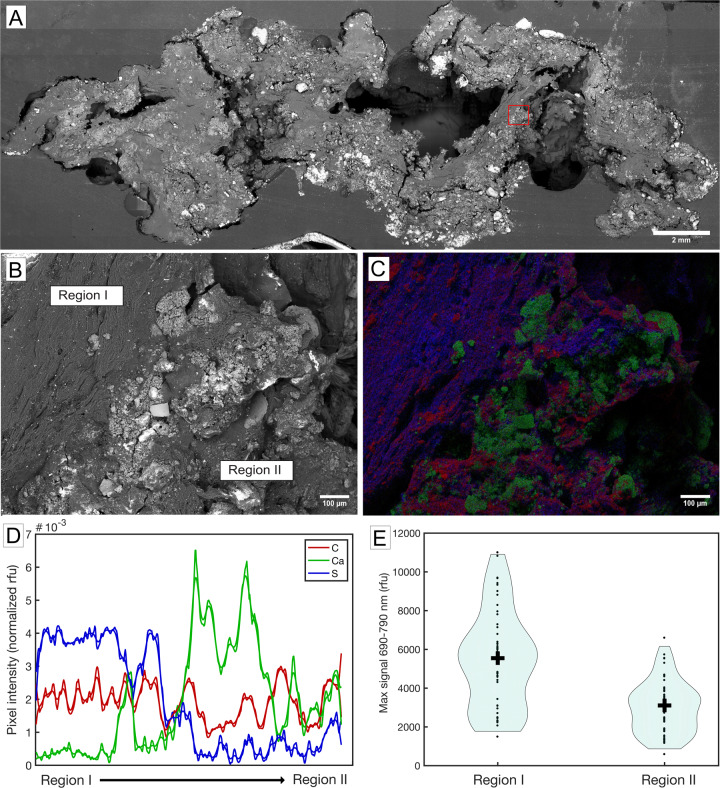
Distributions of carbon, calcium, and sulfur in a pustular mat. **A** SEM image of pustular mat shown in Fig. 1D. The red box marks the region analyzed in panels **B**, **C**; **B** SEM closeup of the region marked by red box in panel **A**; **C** EDS map of carbon (red), calcium (green), and sulfur (blue) of the same region and **D** quantified across Region I and Region II as indicated in panel **B**. **E** Confocal imaging comparing the chlorophyll autofluorescence signal in Region I and Region II.

Cross sections of the original pustular mat were obtained as follows: the mat was collected in the field and preserved at 4 °C in the dark. This sample was fixed in 2.5% glutaraldehyde overnight, rinsed with 0.2 mM sodium cacodylate buffer, submerged for 10 min in a phosphate-buffered saline solution that contained 30% sucrose by weight, and air-dried overnight at 21 °C. The fixed and dried mat was transferred to a weigh boat lined with aluminum foil and submerged in Buehler EpoxiCure 2 Resin (#203430064) and hardener (#203432016) mixed in a 100:23 ratio by weight and left to dry overnight. Bulk sample cuts were made with an acetone-cleaned diamond saw blade (MK Diamond 156651) mounted on a modified tile saw (SKILSAW 3550-02). Fine cross-sections were made with a precision low-speed sectioning saw at 100 rpm (Allied Techcut 4X) at the Harvard University Center for Nanoscale Systems (CNS). The section was imaged by a stereo microscope with a 10 Mpixel camera (Amscope). The final mosaics contain ~20 images that were digitally stitched in Adobe Photoshop (Fig. 1D). A scanning electron micrograph (SEM) of the section was obtained at the Jet Propulsion Laboratory (JPL) (Fig. 2A). The sample was affixed directly to an SEM mounting stage with double-coated carbon conductive tape (Ted Pella Inc., Product #16084-7, Redding, CA, USA) and analyzed uncoated under variable pressure mode at 30 Pa, 15 keV accelerating voltage, and a working distance of 10 mm. A JEOL 7900 F SEM/EDS system and AZtec Software version 5.1 (Harvard University CNS) were used for high resolution mapping and elemental analyses (Fig. 2B, C, Supplementary Fig. 1A). The SEM-EDS images show maps of carbon, calcium, and sulfur intensities measured in each pixel within the region of interest (ROI) (Supplementary Fig. 1B). Figure 2D reports the medians of values along the x-axis of this ROI for each pixel along the y-axis. Trends shown in Fig. 2C–E are representative of three regions of the mat that were analyzed in the same fashion.

### Confocal image acquisition and analysis

A Leica TCS SP5 confocal microscope was used to investigate the chlorophyll autofluorescence signal in the ROIs previously analyzed by SEM/EDS (Fig. 2D). A 543 nm laser line was used as the excitation source and the autofluorescence signal was analyzed by performing a lambda scan from 548 to 791 nm with a bin size of 7 nm (33 bins total). To validate the confocal fluorescence signal against the topographic changes within the ROI, we acquired ~30 z-stacks to cover sufficient topological range. The maximum intensity signal in each bin was used to reconstruct the chlorophyll autofluorescence signal as described previously [32, 33]. The strongest chlorophyll autofluorescence signal within 690–790 nm from each z-stack was selected to create the violin plot and compare the signals in Region I and Region II (Fig. 2E).

### Cyanobacterial enrichment cultures

Pustular mats were grown in sterile plastic plant culture jars (BioExpress, 190 mL, 68 mm × 68 mm) exposed to 24 h of light at 21 °C. The hypersaline modified BG11 medium was replaced every two weeks to maintain the solution pH between 7.5 and 8.5. To enrich pustule forming cyanobacteria from these mats, we repeatedly transferred cyanobacterial pustules between Petri dishes that contained hypersaline BG11 solidified by agar (10.17504/protocols.io.bkchkst6) and hypersaline BG11 liquid medium.

### EPS extraction and characterization

To characterize the cyanobacterial EPS and confirm the presence of sulfate groups, EPS was extracted from dime-sized portions of the pustular mat and cyanobacterial enrichment cultures [17]. The samples were pelleted by centrifugation for 1 min at 4000 rpm, immersed in 2.5 mL of 0.25 M NaCl solution, incubated at 60 °C overnight, and centrifuged for 1 min at 4000 rpm. The supernatants were then transferred to equal volumes of ice-cold 200 proof ethanol in sterile test tubes and the mixtures were incubated at 4 °C overnight. The gelatinous extract from the solution was pelleted by centrifuging for 5 min at 8000 rpm. The resulting pellets were air-dried overnight in a biosafety hood. Fourier transform infrared spectra (4000 cm^−1^ to 600 cm^−1^) of cyanobacterial EPS pellets were acquired on a Bruker Lumos FT-IR Microscope at the Harvard University CNS. Spectra from several spots per sample were processed using Opus Spectroscopy Software. EPS samples extracted from the pustular mat matrix were analyzed by a Thermal Scientific Nicolet™ iS50 FTIR Spectrometer at the MIT Materials Research Laboratory.

A modified toluidine blue assay confirmed the presence of sulfated polysaccharides in the extracted cyanobacterial EPS using a recently updated method [34]. Standard solutions of non-sulfated carbohydrates, a negatively charged non-sulfated polysaccharide (xylan), and a sulfated polysaccharide (fucoidan ≥95% Sigma-Aldrich F8190-500MG) were dissolved in milliQ water at concentrations of 0.25 g/L and 1.5 g/L and sterilized by filtration through 0.2 µm Pall Acrodisc® Sterile Syringe Filters with Supor® Membrane (VWR, Radnor, PA, USA, catalog # 28143-350). Samples of extracted EPS (~17 mg dry weight) from the cyanobacterial enrichment cultures were diluted with 100 µL of milliQ water. 100 µL of each sample and standard were transferred to 1.5 mL Eppendorf^®^ microtubes (Eppendorf North America, NY, USA, cat#022364111) and mixed with 900 µL of 0.06 mM toluidine blue reagent in 0.02 M maleic acid buffer (pH 1). 200 µL aliquots of these solutions were added to separate wells of a 96-well plate and their absorbances between 400 and 700 nm measured using a BioTek microplate reader (BioTek, Synergy 2, Winooski, VT, USA). The spectra were analyzed by BioTek Gen5 Data Analysis software.

### MAG sequencing, assembly, and binning

Whole genomic DNA was extracted from the original enrichment cultures of pustular mats using a Powersoil^®^ DNA Isolation Kit (MO BIO Laboratories, Inc Carlsbad, CA, USA). A Qubit 2.0 fluorometer (Thermo Fisher Scientific, Chino, CA, USA) was used to measure DNA yield. DNA purity was evaluated using a NanoDrop 2500 spectrophotometer (Thermo Fisher Scientific, Chino, CA, USA). The metagenomes were sequenced at BioMicro Center Core Facility at the Massachusetts Institute of Technology using an Illumina NextSeq sequencer. DNA sequence fragments were quality filtered and trimmed using Trimmomatic v.0.36 (ref. [35]). Resulting reads were assembled using MEGAHIT v1.0.2. and binned using MetaBAT v2.12.1 (refs. [36–38]).

### Quality control, taxonomy assignment, and MAG annotation

The quality and completeness of sequenced and assembled genomes were assessed using the CheckM pipeline [39]. MAGs that did not meet a > 50% completeness and <10% contamination threshold were not included in the dataset. MAGs that met quality standards were assigned taxonomic classifications using GTDB-Tk v0.2.2 (ref. [40]) and annotated using the Department of Energy (DOE) Joint Genome Institute Integrated Microbial Genomes (JGI IMG) Annotation Pipeline v.4.16.5 (refs. [41, 42]). Supplementary Table 1 summarizes the taxonomic assignments and CheckM outputs for all MAGs that met quality standards. Figures include GTDB-Tk taxonomy naming, but NCBI taxonomic naming will be used for phyla and class throughout the text (Supplementary Table 1). All metagenomes are available on JGI under GOLD AP ID Ga0316160. Raw sequence data and MAGs are also publicly available on Zenodo (10.5281/zenodo.3874996).

### Phylogenomic tree

A phylogenomic tree was created using the anvi’o (v5.5) workflow for phylogenomics [43]. An anvi’o contigs database was created using the MAGs that met quality standards. The Campbell et al. HMM profile was used to identify genes for concatenation [44]. The following ribosomal proteins were used for phylogenomic analyses: Ribosomal L1, Ribosomal L2, Ribosomal L3, Ribosomal L4, Ribosomal L5, and Ribosomal L6. A phylogenomic tree was generated using the concatenated amino acid sequences and visualized in the anvi’o interactive interface. GTDB-Tk taxonomy and completeness information was overlaid onto the tree. MAG 78 was removed due to inconsistencies between its phylogenomic placement based on ribosomal proteins and taxonomic assignment based on GTDB-Tk taxonomy. A reproducible workflow for the reconstruction of this phylogenomic tree can be found in the following GitHub repository: https://github.com/emilieskoog/SharkBay2020-analysis/tree/master/PhylogenomicTree.

### Mapping the genes that encode for the production and degradation of sulfated EPS in MAGs

Each MAG was evaluated for the presence and absence of genes involved in the production and degradation of sulfated EPS. On the production side, we focused on sulfotransferase, glycosyltransferase, and polysaccharide export genes because all have established and characterized roles in the synthesis and export of sulfated polysaccharides: sulfotransferases catalyze the addition of a sulfate group to a carbohydrate residue [45] and glycosyltransferases establish glycosidic linkages in polysaccharides.

To understand the potential to degrade sulfated EPS, we analyzed each MAG for formylglycine-dependent sulfatases, formylglycine-generating enzymes (FGE), and glycosyl hydrolases. Formylglycine-dependent sulfatases require a FGE to produce formylglycine for catalysis [46] and constitute the largest family of sulfatases that catalyze the removal of sulfate groups from various molecules including polysaccharides [29]. MAGs were also analyzed for the presence of homologs of the novel, non-formylglycine-dependent carrageenan sulfatase of *Pseudoalteromonas carrageenovora* [47]. This sulfatase is in a novel, non-formylglycine-dependent family and is known to act on marine sulfated polysaccharides. By expanding the search to include homologs of this novel family, we can better understand the capacity for this microbial community in degrading sulfated polysaccharides using genes outside of the more common formylglycine-dependent sulfatases. Analyses also included several members from the large families of glycosyltransferases and glycosyl hydrolases, enzymes involved in the synthesis and breakdown of specific oligo- and polysaccharides, respectively.

A BLAST database was constructed with all 83 quality-filtered MAGs. Several different PFAM and conserved domains for sulfotransferase (PF13469), formylglycine-dependent sulfatase (PF00884), FGE (PF03781), the novel *P. carrageenovora* carrageenan sulfatase (GenBank: AER35705.1), and polysaccharide export (cd00267) genes were selected as query sequences to search for within each MAG database using BLAST v.2.6.0+. Because there are many different subfamilies of glycosyltransferase and glycosyl hydrolase genes that can act on different substrates, JGI IMG annotations were first used to identify the presence of each unique glycosyltransferase and glycosyl hydrolase PFAM hit present within each MAG. Each of these PFAM hits was then used as a query sequence to identify the e-value and percent identity of each glycosyltransferase and glycosyl hydrolase gene using BLAST v.2.6.0+. Hits that had an e-value of at least 1e^−10^ and at least 30% identity to the protein query were accepted for all genes of interest. This percent identity was accepted because sulfotransferase and sulfatase sequence queries are largely derived from eukaryotic genomes, especially human, and are expected to be less similar to those present in bacterial MAGs. A presence/absence heatmap was generated using ggplot2 in R (v.3.6.0) to show the percentage of MAGs that contained at least one copy of each of the queried genes involved in the respective pathways of production and degradation of sulfated polysaccharides within each taxonomic order. A reproducible workflow can be found here: https://github.com/emilieskoog/SharkBay2020-analysis/tree/master/Presence:Absence_Plot.

### Sulfatase activity

Sulfatase activity was determined by the colorimetric Sulfatase Activity Assay Kit (Sigma-Aldrich, MO, USA, cat# MAK276). Triplicates of dime-sized portions of cyanobacterial enrichments and pustular mats rehydrated in the modified BG11 hypersaline medium were either manually shaken or placed on ice and homogenized with a SONIFIER 150 ultrasonic cell disrupter and homogenizer (maximum power 180 W) at 22.5 kHz for 20 min. The sonicated samples were centrifuged at 10,000 g for 10 min at 4 °C after the addition of 25 µL of Promega Protease Inhibitor Cocktail (50X) (Promega Co., Madison, WI, USA, cat# G6521). 10 µL of these supernatants were placed in separate wells of a 96-well polystyrene plate and mixed with a reaction mix that contained 50 µL sulfatase assay buffer and 40 µL sulfatase substrate. The background control consisted of 10 µL of modified BG11 hypersaline medium and 90 µL of sulfatase assay buffer and the plates were incubated at 37 °C for 30 min. All samples and background controls were analyzed in triplicate. The positive controls and standard curve were prepared as described by the assay manufacturer. After incubation, 100 µL of Stop/Developing Solution was added to each well. Each well was mixed and absorbance was measured at 515 nm on a BioTek microplate reader.

To convert nmoles of 4-nitrocatechol generated during the 30-minute reaction time to sulfatase activity, we used a Thermo Scientific™ Pierce™ Coomassie (Bradford) Protein Assay kit (ThermoFisher Scientific, NY, USA, cat# PI23200). Two standard series of bovine serum albumen (BSA) for a working range of 1–25 µg/mL and 100–1500 µg/mL were prepared in milliQ water according to the manufacturer’s protocol. Triplicates of sonicated and unsonicated cyanobacterial enrichment and mat samples were prepared for each working range and added to a 96-well microplate. The plate was shaken for 1 min, incubated at room temperature for 10 min, and the absorbances of samples and standards were measured at 595 nm. All absorption values were averaged and sulfatase activity was determined according to the manufacturer’s protocol.

### Extracellular and periplasmic sulfatases

We analyzed the amino acid sequences of sulfatases annotated in JGI IMG from a few MAGs from each order that contained all of the genes necessary for the degradation of sulfated EPS. These sequences were submitted to the SignalP 5.0 online server (https://services.healthtech.dtu.dk/service.php?SignalP-5.0) to assess whether these annotated sulfatases were likely intracellular or periplasmic [48].

## Results

### Sulfur gradient in pustular mats

SEM/EDS analyses of pustular mats revealed a strong sulfur signal throughout the mat sections. Sulfur was more abundant in the regions that exhibited a significantly stronger (*p*-value < 0.005, two sample two-sided t-test) chlorophyll autofluorescence signal within EPS (Region I) compared to areas that contained EPS and carbonate minerals (Region II), which contained a lower chlorophyll autofluorescence signal (Fig. 2; Supplementary Fig. 1A). These gradients indicated a correlation between processes that incorporate sulfur in EPS and areas where photosynthetic pigments and cyanobacteria are more abundant.

### Sulfated EPS in pustular mats and cyanobacterial enrichments

**Fig. 3:**
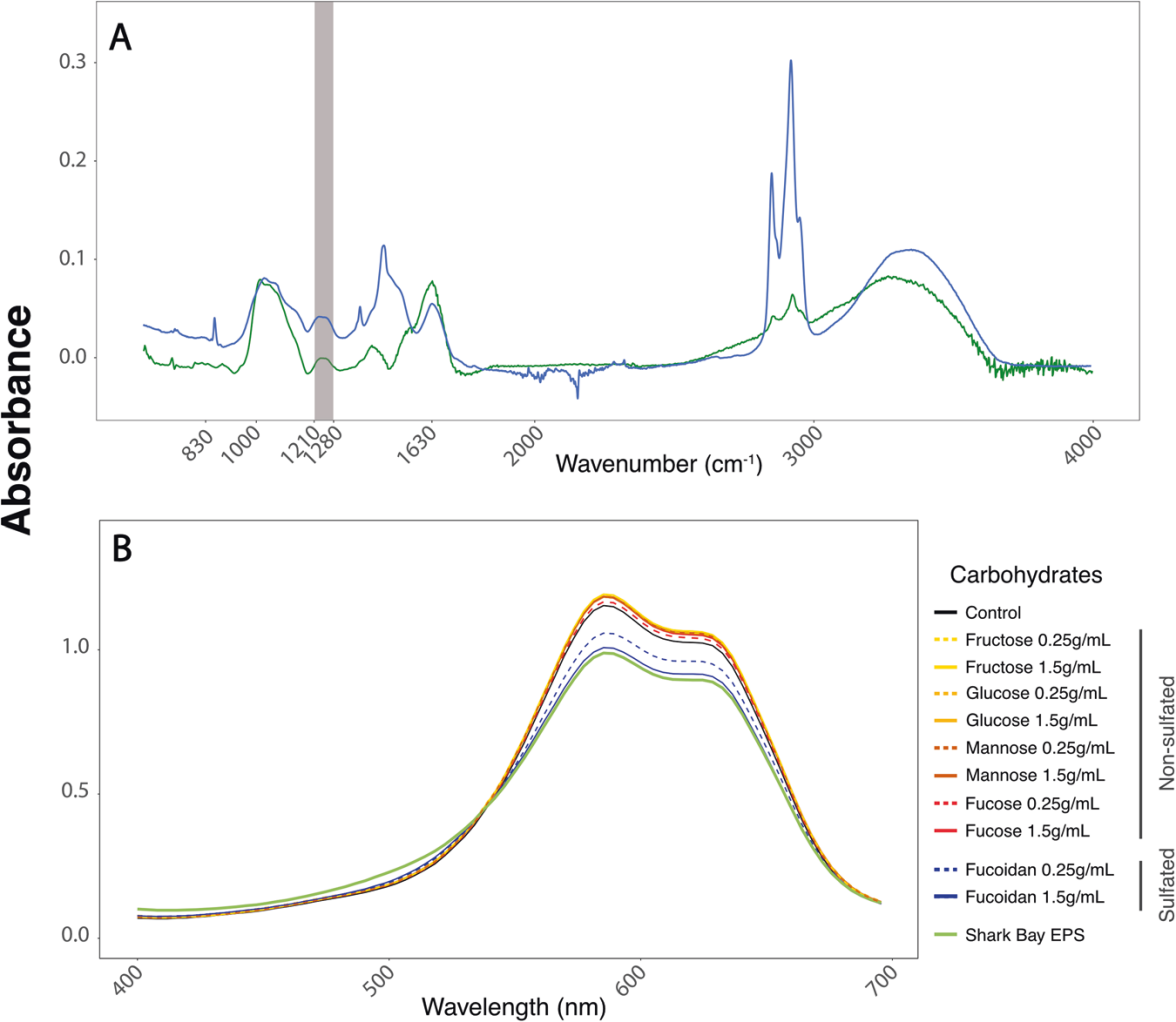
Analyses of extracted EPS. **A** FT-IR analysis of Shark Bay cyanobacterial enrichment EPS (green) and EPS from pustular mat matrix (blue). **B** Toluidine blue assay [34] of non-sulfated carbohydrates (warm colors), sulfated polysaccharides (blue), and EPS extracted from pustular mats enriched from Shark Bay (green). Solid and dashed lines show higher and lower concentrations of individual compounds, respectively. Sulfated polysaccharides exhibit a decreased absorption in the 550–650 nm region due to the formation of ﻿toluidine blue-sulfated polysaccharide complexes and a decreased absorption by the α- and β-bands of the toluidine blue monomers and dimers, respectively.

To verify that the cyanobacteria produced sulfur-containing EPS, we analyzed the EPS extracted from the natural, unenriched mat and the EPS produced by mat cyanobacterial enrichment cultures using Fourier transform infrared spectroscopy (FT-IR). FT-IR spectra of two different points within the cyanobacterial EPS contained small triple peaks between 805 and 840 cm^−1^, indicative of the sulfation of specific galactose units [[49]; Fig. 3A]. All samples also exhibited the broad band (shaded) between 1210 and 1280 cm^−1^ indicative of the S═O stretching vibration of sulfate groups ([49]; Fig. 3A) and peaks at 1050 cm^−1^ that can be attributed to the stretching vibrations of the RO─SO_3_^−^ bond of sulfate groups [49]. These spectroscopic analyses confirmed the presence of sulfate groups in EPS. Additional strong peaks at ~1630 cm^−1^ corresponded to the stretching vibration of the carbonyl group of carboxylic acid groups (C═O) [49]. Small peaks at 740 and 716 cm^−1^ were attributed to C─O─C bending vibrations indicative of glycosidic linkages in polysaccharides [49].

To confirm the presence of sulfated polysaccharides in cyanobacterial pustules, we used a specific toluidine blue colorimetric assay (Fig. 3B; Supplementary Fig. 2, [34]). The spectra of EPS extracted from cyanobacterial enrichment cultures were compared to those of four non-sulfated carbohydrates, the non-sulfated polysaccharide xylan (Supplementary Fig. 2), and fucoidan, a sulfated polysaccharide. The absorbance peak maxima in these spectra decrease with the increasing sulfation and concentrations of sulfated polysaccharides ([34], Supplementary Fig. 2). Fucoidan and the EPS extracted from active enrichment cultures of pustule-forming cyanobacteria from Shark Bay [17] exhibited the same decrease in absorbance peak maxima (Fig. 3B), indicating the presence of sulfated polysaccharides in the latter.

### Community structure

**Fig. 4:**
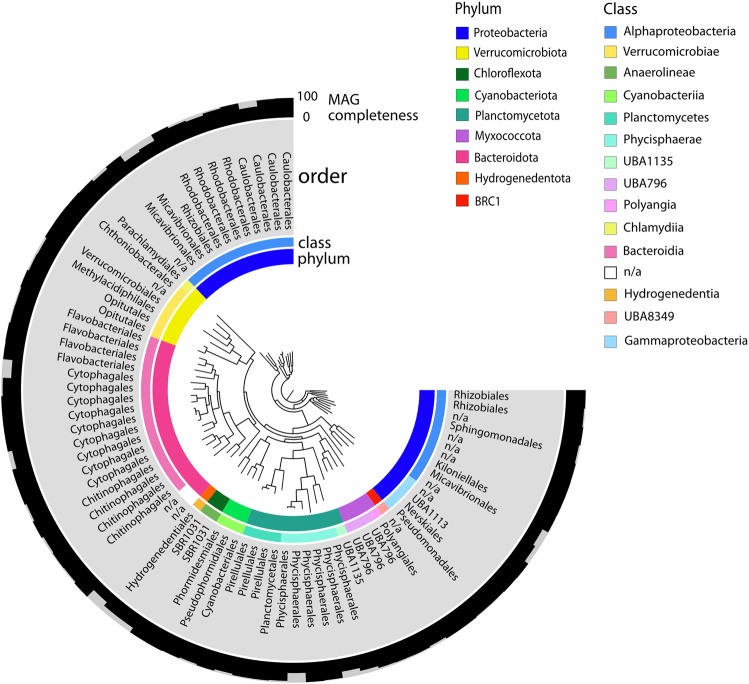
Phylogenomic community analysis. Phylogenomic tree of MAGs from pustular mats. GTDB-Tk taxonomy is shown in the inner ring (phylum), the middle ring (class), and the outer ring (order). The outermost ring visualizes the completeness of individual MAGs from 0–100%. Only the 83 MAGs that passed quality tests (>50% completeness; <10% contamination) are included.

Next, we sequenced the genomic DNA extracted from the original enrichment cultures of pustular mats, assembled nearly 120 MAGs of varying quality, and analyzed them to verify the cyanobacterial potential to produce sulfated polysaccharides. These analyses also enabled us to assess the potential of other organisms to metabolize these compounds. We recovered 84 MAGs after quality control (>50% completeness; <10% contamination) of the entire sequenced microbial community. Nearly 70% of these MAGs were >90% complete and <5% contaminated (Fig. 4; Supplementary Table 1), enabling the inferences based on the presence and absence of genes within the MAGs. Of these, MAG 78 was removed because its phylogenetic placement was inconsistent with its taxonomic assignment, leaving the 83 MAGs discussed in this study. Many of the assembled MAGs belonged to the Proteobacteria phylum (40%), with 80% Alphaproteobacteria and the remaining 20% Gammaproteobacteria (Fig. 4). Bacteroidetes comprised ~24% of MAGs, Planctomycetes ~13%, and Verrucomicrobia nearly 10%. Approximately 7% of the MAGs were identified as Myxococcota, Hydrogenedentes, and BRC1. Two Chloroflexi MAGs and three Cyanobacteria MAGs were also recovered. Each of the three cyanobacterial MAGs was >90% complete and <1.3% contaminated. The community composition of recovered MAGs was consistent with the previously reported composition based on 16 S rRNA sequences: the higher abundances of Proteobacteria (40.8% species-level specific operational taxonomic units (OTUs)), Bacteroidetes (12.8% OTUs) and Planctomycetes (12.1% OTUs), and low abundances (4.1% OTUs) of cyanobacteria [30, 50, 51]. The general consistency between community compositions based on MAGs from rehydrated, illuminated mats and 16S rRNA amplicons from natural samples indicates the recovery of representative communities and genomic information. The different abundances of some less abundant groups compared to the previously reported abundances [51] may indicate the lesser extent of anaerobic conditions during rehydration and issues with sampling the surface and deeper parts of irregularly shaped pustular mats.

### Functional potential for the production of sulfated EPS in pustular mats

Cyanobacteria accounted for fewer than 10% of all MAGs, but two of the three Cyanobacteria MAGs—Phormidesmiales and Pseudophormidiales—contained sulfotransferase, glycosyltransferase, and polysaccharide exporter genes, all necessary for the production of sulfated EPS (Supplementary Fig. 3). Both of these cyanobacterial MAGs contained a ~20,000-nucleotide-long region of genes with predicted functions in biosynthesis of sulfated polysaccharides (Supplementary Fig. 3) and polysaccharide modification (Supplementary Table 2) [52, 53]. The presence of glucose dehydrogenase and specific fucose acetyltransferase genes only in the *Phormidesmiaceae* MAG and heparinase and mannose dehydrogenase genes only in the *Pseudophormidaceae* MAG (Supplementary Table 2) suggested that these two cyanobacteria produced sulfated polysaccharides that contain different mono- and polysaccharide units.

**Fig. 5:**
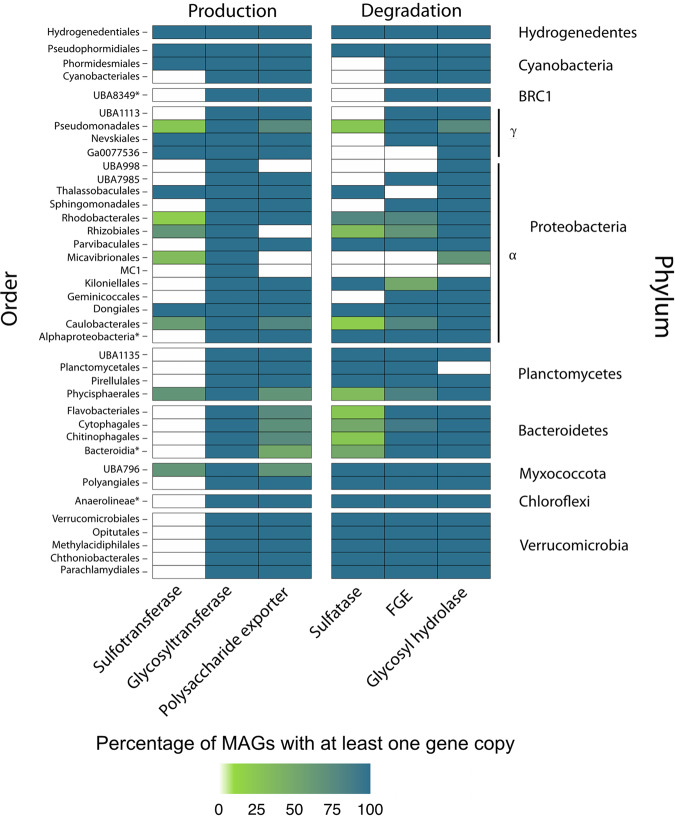
Presence/absence of genes involved in the production or degradation of sulfated polysaccharides. Color bars indicate the percentage of MAGs within each order that contain each of the genes involved in the production and degradation of sulfated polysaccharides. Production: sulfotransferase, glycosyltransferase, polysaccharide exporter genes; degradation: formylglycine-dependent sulfatase, formylglycine-generating enzymes (FGE), and glycosyl hydrolase genes. Shark Bay MAG orders are classified using GTDB-Tk. *Taxonomy only defined at the class level.

A separate blasting protocol also supported the potential to produce sulfated polysaccharides in two out of three sequenced Cyanobacteria MAGs (Fig. 5). We found at least one copy of each of the genes necessary for the production of sulfated polysaccharides in 100% of the representative MAGs from more than 12% (5/39) of all orders. These included Hydrogenedentiales, Nevskiales (Gammaproteobacteria), Ga0077536 (Gammaproteobacteria), Thalassobaculales (Alphaproteobacteria), and Dongiales (Alphaproteobacteria). Some MAGs in a few orders within the Proteobacteria, Myxococcota, and Planctomycetes phyla also contained the sulfotransferase gene (Fig. 5), but all BRC1, Bacteroidetes, Chloroflexi, and Verrucomicrobia MAGs and most orders in the Planctomycetes phylum lacked this gene (Fig. 5).

### Potential for degradation of sulfated polysaccharides

Sulfated EPS produced by cyanobacteria and other microbes may be metabolized by other microorganisms in pustular mats. We tested this hypothesis by identifying the percentage of MAGs within each order that contained at least one copy of each of the major genes involved in the degradation of sulfated polysaccharides (glycosyl hydrolase, formylglycine-dependent sulfatase, and FGE) and, separately, homologs of the *P. carrageenovora* novel carrageenan sulfatase gene (see Methods). All Hydrogenedentes, Myxococcota, Verrucomicrobia, Chloroflexi, and a majority of the Planctomycetes MAGs contained all three genes necessary for the degradation of sulfated EPS (Fig. 5). All MAGs within the Parvibaculales and Dongiales orders and another undefined order within Alphaproteobacteria also contained each of the three genes required to degrade sulfated EPS, as did one Cyanobacteria MAG (Fig. 5). A fraction of MAGs from each Bacteroidetes order contained all three degradation genes, as did a few MAGs within the Pseudomonadales, Rhodobacterales, Rhizobiales, Kiloniellales, and Caulobacterales orders (Fig. 5). Sequence homologs of the *P. carrageenovora* novel carrageenan sulfatase gene were identified in 6 Bacteroidetes, 2 Planctomycetes, and 4 Proteobacteria MAGs, 75% of which lacked a formylglycine-dependent sulfatase. Thus, the ability to remove sulfate from sulfated polysaccharides was widely distributed in the MAGs from pustular mats. The predicted localization of sulfatase proteins produced by some sulfatase-containing MAGs from all phyla found that some sulfatases should be excreted by the Sec or Tat translocon, while others should reside in the periplasm. All Cyanobacteria MAG sulfatase proteins were predicted to contain signal peptides indicating that they are transported extracellularly by the Sec or Tat translocon, in keeping with the need to modify their multilayered extracellular envelopes that extend over multiple cell diameters. In contrast, MAGs from all other phyla contained both extracellular and periplasmic sulfatases.

Sulfatase activity was confirmed in unsonicated and sonicated samples of cyanobacterial pustules and a homogenized Shark Bay mat (included non-cyanobacterial EPS, cyanobacterial EPS, and sediment) using a sulfatase assay. The highest activity, ~120 mU/mg protein (nmole/min/mg), was present in cyanobacterial pustules that had not been sonicated. Sulfatase activity was about 33% lower in the samples of unsonicated mats. This suggested the association of sulfatase activity with fresh cyanobacterial EPS (Supplementary Fig. 4) both in the natural mats and the enrichments of pustule-forming cyanobacteria.

## Discussion

### Sulfated polysaccharides in microbial mats

Some marine algae, sulfate reducing bacteria, and cyanobacteria are known to produce sulfated polysaccharides in culture [17, 18, 26, 54–56], but Shark Bay pustular mats were not reported to contain these compounds. In fact, studies of biomineralizing microbial mats in hypersaline environments typically do not consider the potential presence of sulfate moieties and instead focus on carboxylic, phosphate, amine, and sulfide groups in EPS [57, 58]. Sulfated polysaccharides have been previously identified in microbial mats from Polynesian “kopara” [59, 60] and atolls [61] and Bahamian microbial [28] mats. The latter study attributed the production of sulfated EPS to sulfate reducing bacteria and considered the potential of these compounds to bind calcium [28]. However, sulfate reducing microbes are not reported as abundant in Shark Bay pustular mats [51] and are thus unlikely to be the major producers of sulfated EPS. The analyses presented here reveal abundant sulfate in the EPS matrix of cyanobacterial pustules and peritidal pustular mats from Shark Bay, map sulfur onto the areas that contain a higher signal of photosynthetic pigments, and point to cyanobacteria as the main, but not only, producers of sulfated EPS in this hypersaline microbial system (Figs. 2,  3,  5).

The demonstrated protective and hygroscopic roles of sulfated polysaccharides in marine algae suggest similar functions for these polysaccharides in the pustular mats from Shark Bay. For example, the upregulation of sulfotransferase genes involved in the production of sulfated polysaccharides by some microalgae has been linked to water absorption and retention during desiccation events [62]. Sulfated polysaccharides extracted from algae have also been shown to exhibit antioxidant and free radical scavenging activities [63, 64]. The negative charge of sulfate groups in sulfated polysaccharides are also hypothesized to protect the community against viral infection [65, 66].

### Degradation of sulfated EPS

The widespread presence of genes involved in the removal of sulfate from polysaccharides in MAGs and the detectable sulfatase activity within our samples provide evidence for the modification and degradation of sulfated EPS in pustular mats. Many microbial groups with the functional potential to degrade EPS in the pustular mats have also been shown to degrade sulfated polysaccharides across multiple environments including within marine algal-derived sulfated EPS matrixes [67–71]. These include fucoidan-degrading Verrucomicrobia [72, 73] and several lineages of Planctomycetes shown to metabolize fucoidan and carrageenan produced by brown and red algae [74, 75] or hypothesized to do so [76]. *Anaerolineae* enriched from extracellular matrixes of marine sponges have also been reported to contain arylsulfatases and other genes involved in the metabolism of sulfated polysaccharides [77, 78]. Some Bacteroidetes strains have been shown to break down sulfated glycosaminoglycans within the human gut [79]. Our analyses also reveal the potential for Hydrogenedentes to degrade sulfated polysaccharides although this function was not previously recognized in this phylum.

Sulfate is an abundant anion in seawater but is not a dominant terminal electron acceptor in pustular mats [30, 51, 80]. Instead, sulfate ester groups in the EPS of pustular mats can influence the heterotrophic degradation of these compounds: multiple studies suggest that the degradation and utilization of certain sulfated polysaccharides requires the removal of sulfate to form neutral mono- and oligosaccharides that can be used in catabolic reactions [29, 81–86]. In particular, the sulfatases excreted by some microbes to break down sulfated EPS outside of the cell may make carbohydrates bioavailable to organisms that do not contain sulfatases, fueling microbial interactions centered around sulfated polysaccharides. The predicted presence of both periplasmic and extracellular sulfatases in sulfatase-containing MAGs from all phyla other than Cyanobacteria points to the potential roles of many of these microbes in such partnerships.

**Fig. 6:**
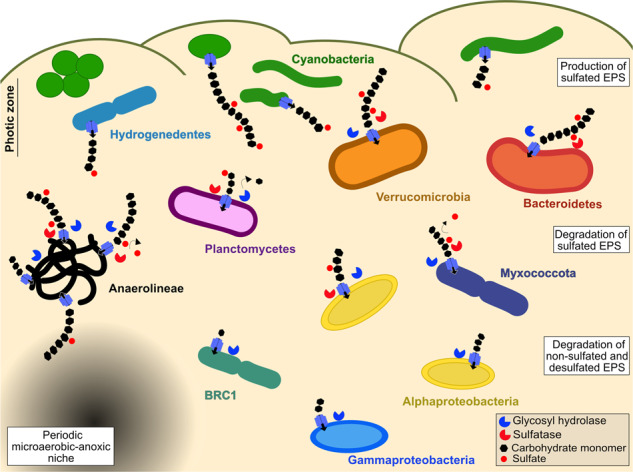
Hypothesized distribution of organisms at and away from the surface of a pustular mat. The schematic shows microbial groups represented by MAGs with inferred potentials to produce sulfated EPS and degrade sulfated, desulfated, and non-sulfated EPS.

The removal of sulfate during the degradation of EPS is consistent with the observed gradients of sulfur abundance (Fig. 2) and the previously established distribution of microbial phyla in pustular mats based on 16S rRNA sequencing [51]. Cyanobacteria, Bacteroidetes, and Alphaproteobacteria are the dominant phyla within the surface layer (0–2 mm), Verrucomicrobia are present in the same layer, Alphaproteobacteria are present throughout the mat, and Gammaproteobacteria are present at lower mat depths (i.e., further from cyanobacteria) [51]. Figure 6 outlines some hypotheses about the roles of organisms from these phyla in the production and degradation of sulfated polysaccharides in pustular mats. The confirmed production of sulfated polysaccharides by Cyanobacteria is consistent with the higher abundance of sulfur in the areas of mats that contain stronger chlorophyll autofluorescence (Fig. 2). The spatial proximity of Verrucomicrobia and Bacteroidetes to Cyanobacteria, as reported by Wong et al. (2015), is consistent with the here-identified functional potentials of multiple orders of Verrucomicrobia and Bacteroidetes to degrade sulfated EPS produced by Cyanobacteria. Most Gammaproteobacteria encode the capacity to degrade desulfated polysaccharides and only few MAGs in the Pseudomonadales order have the capacity to degrade sulfated polysaccharides. Indeed, Gammaproteobacteria are reported as more abundant in the deeper areas of the mat [51], away from Cyanobacteria, the most likely source of sulfated EPS (Fig. 2). Our results also suggest that some Pseudomonadales may be more common in the regions where sulfated EPS abounds.

### Calcium carbonate precipitation and implications for preservation in the rock record

Calcium carbonate precipitates in natural pustular mats occur primarily in cyanobacteria-poor regions where sulfur is depleted (Fig. 2). These geochemical gradients match some previous inferences about the dependence of microbial carbonate precipitation on the properties of EPS [9, 10, 14, 16, 28, 87, 88]. In microbialites from hypersaline ponds in Kiritimati (Kiribati), fresh cyanobacterial EPS was suggested to inhibit carbonate nucleation and lead to the formation of sparse ooid nuclei, whereas the more degraded EPS enables more extensive carbonate precipitation [87, 88]. In fact, the fresh, sulfur-rich EPS produced by pustule-forming cyanobacteria have an affinity for magnesium cations over calcium cations and contribute to the precipitation of Mg-rich amorphous silica rather than carbonate [8, 17]. In contrast, sulfated EPS produced by some modern sulfate reducing bacteria have a high affinity for calcium and have been implicated in the precipitation of aragonite and high-Mg calcite in Bahamian microbial mats [28]. However, given that sulfate reducing bacteria are not very abundant in any zones of pustular mats [51], they are unlikely to be major contributors to sulfated EPS. Based on this, we hypothesize that cyanobacterial sulfated EPS may inhibit carbonate nucleation, whereas the desulfation and concomitant degradation of EPS in cyanobacteria-poor zones promotes carbonate precipitation. The identification of genes involved in the production and cycling of sulfated EPS in pustular mats, the demonstrated sulfatase activity, and the geochemical gradients described here enable further tests of this hypothesis. These can include characterizing the diversity of sulfated EPS and genes required to break down unique sulfated and non-sulfated polysaccharides, assessing the degree of sulfation of EPS from the surface to the deeper zones of the mat, quantifying the transcription of specific sulfatases and hydrolases, and mapping the distribution of organisms with now recognized potentials to remove sulfate or degrade the already desulfated EPS.

Here, we assemble one of the largest publicly available datasets of MAGs from a modern peritidal pustular microbial mat, confirm the presence of sulfated polysaccharides in the EPS matrix of these systems, explore the potential cycling of these compounds, and hypothesize their role in microbial community structure, carbon cycling, and precipitation of carbonate minerals.

## Supplementary information

Supplemental Figures

Supplemental Table 1

Supplemental Table 2
