# Supplementary material for: Metagenomic, (bio)chemical, and microscopic analyses reveal the potential for the cycling of sulfated EPS in Shark Bay pustular mats
Source: ISME Commun. 2022 May 19;2:43. doi: 10.1038/s43705-022-00128-1 (PMC9723792; doi:10.1038/s43705-022-00128-1)
Supplement: Supplementary file 1 — Supplemental Figures [file 43705_2022_128_MOESM1_ESM.pdf]

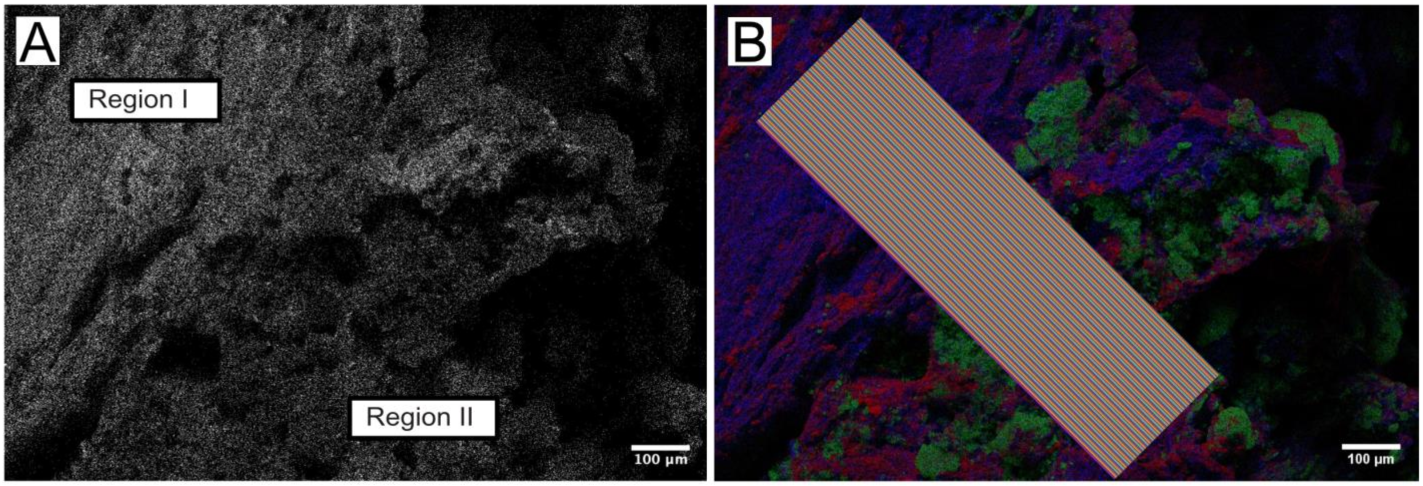

Supplemental Figure 1. SEM-EDS images highlighting sulfur and ROI for mapping elemental change. (A) EDS map of sulfur distribution in the mat region shown in Figure 2B; (B) ROI in Figure 2C used to create the map of intensities in Figure 2D.

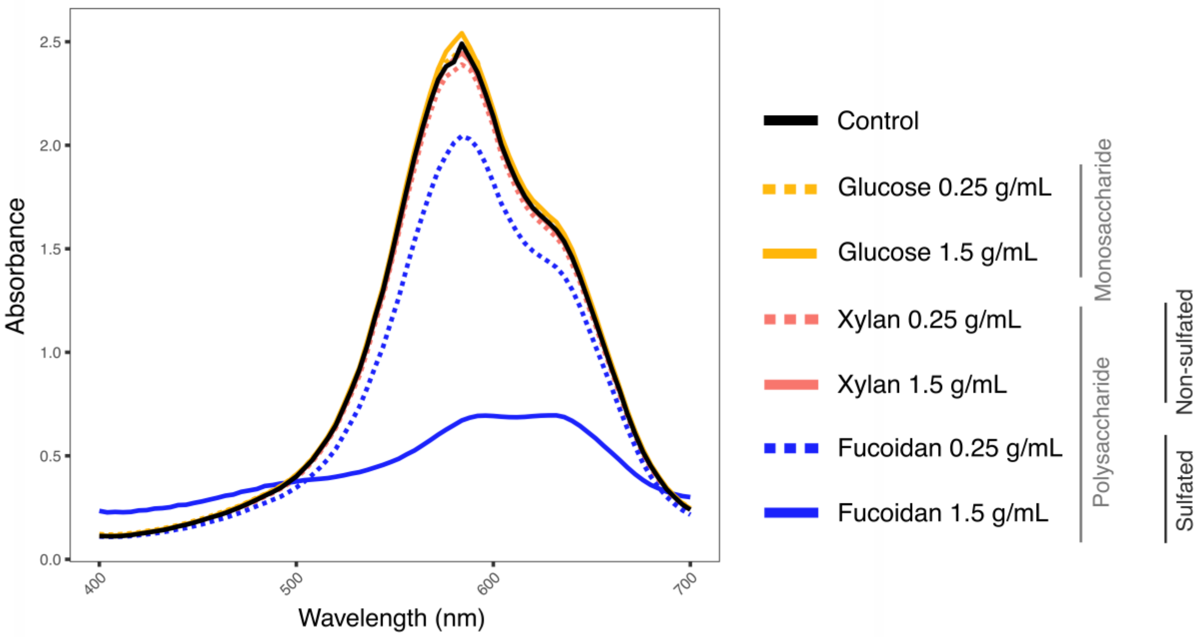

Supplemental Figure 2. Toluidine blue assay of sulfated and non-sulfated polysaccharides. Toluidine blue assay comparing absorbances of sulfated polysaccharides (blue) with non-sulfated monosaccharides (yellow) and polysaccharides (salmon) at varying concentrations (see legend).

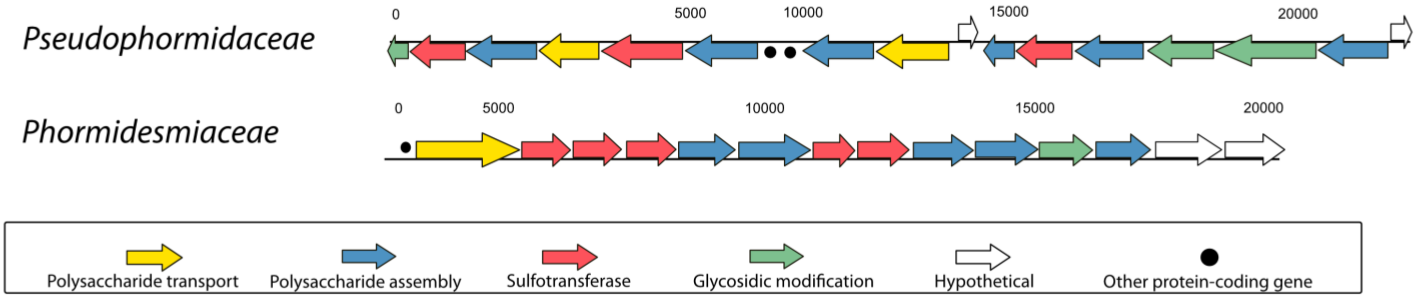

Supplemental Figure 3. Genes with hypothesized roles in the production of sulfated polysaccharides in cyanobacterial MAGs and their neighborhoods. Shown are genes involved in the general assembly, modification, sulfation, and transport/export of polysaccharides. Legend explains the color coding of specific protein-encoding genes within each gene neighborhood. Number markers indicate the length of gene neighborhoods (nucleotides).

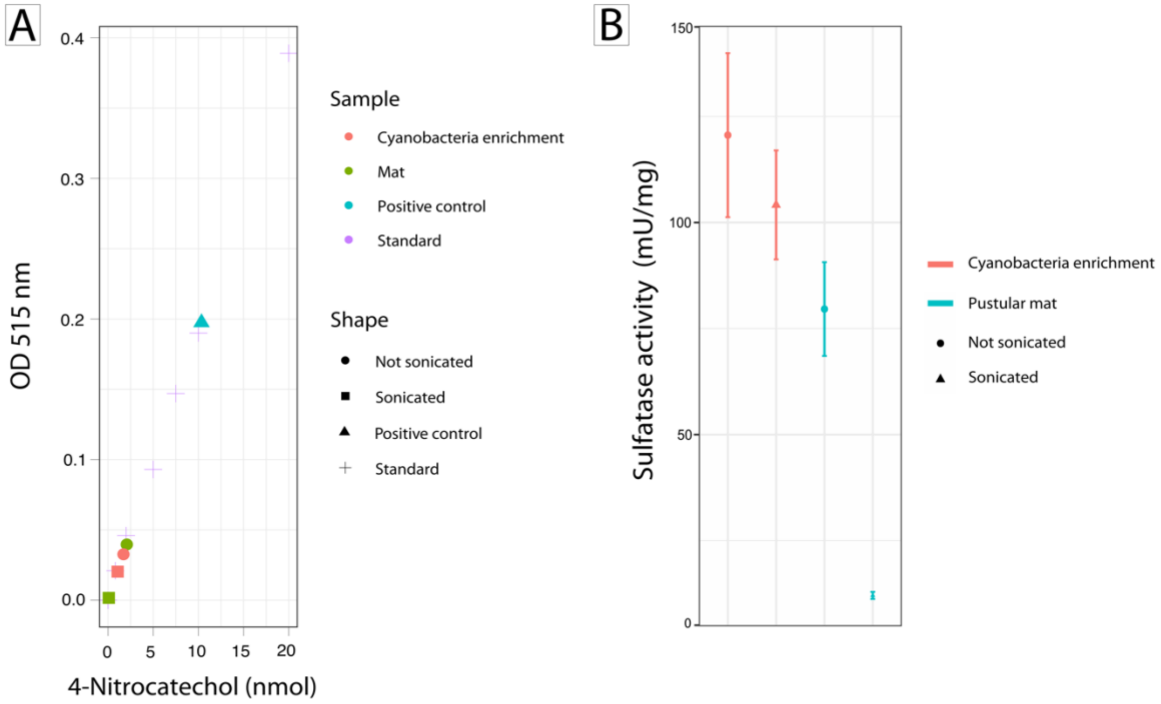

Supplemental Figure 4. Sulfatase activity assay in pustular mats and enriched cyanobacteria. A) 4-Nitrocatechol (nmol) standard curve with nmol of 4-Nitrocatechol generated by sulfatase. B) Quantified sulfatase activity of each sample. Sonication reduced the sulfatase activity in both pustules and mat samples that had not undergone enrichment, likely due to the disruption of extracellular enzymes required to degrade sulfated exopolymers.
